# Single-cell RNA sequencing reveals different chondrocyte states in femoral cartilage between osteoarthritis and healthy individuals

**DOI:** 10.3389/fimmu.2024.1407679

**Published:** 2024-05-29

**Authors:** Zewen Sun, Mingyue Yan, Junjie Wang, Haoyun Zhang, Xiaobin Ji, Yujing Xiao, Tianrui Wang, Tengbo Yu

**Affiliations:** ^1^ Qingdao Medical College, Qingdao University, Qingdao, Shandong, China; ^2^ Department of Orthopedics, The Affiliated Hospital of Qingdao University, Qingdao, Shandong, China; ^3^ Department of Pathology, The Affiliated Hospital of Qingdao University, Qingdao, Shandong, China; ^4^ Department of Orthopaedic Surgery, Qingdao Municipal Hospital, Qingdao, Shandong, China

**Keywords:** osteoarthritis, chondrocyte, single-cell RNA sequencing, inflammation, cell differentiation

## Abstract

**Background:**

Cartilage injury is the main pathological manifestation of osteoarthritis (OA). Healthy chondrocyte is a prerequisite for cartilage regeneration and repair. Differences between healthy and OA chondrocyte types and the role these types play in cartilage regeneration and OA progression are unclear.

**Method:**

This study conducted single-cell RNA sequencing (scRNA-seq) on the cartilage from normal distal femur of the knee (NC group) and OA femur (OA group) cartilage, the chondrocyte atlas was constructed, and the differences of cell subtypes between the two groups were compared. Pseudo-time and RNA velocity analysis were both performed to verify the possible differentiation sequence of cell subtypes. GO and KEGG pathway enrichment analysis were used to explore the potential functional characteristics of each cell subtype, and to predict the functional changes during cell differentiation. Differences in transcriptional regulation in subtypes were explored by single-cell regulatory network inference and clustering (SCENIC). The distribution of each cell subtype in cartilage tissue was identified by immunohistochemical staining (IHC).

**Result:**

A total of 75,104 cells were included, they were divided into 19 clusters and annotated as 11 chondrocyte subtypes, including two new chondrocyte subtypes: METRNL+ and PRG4+ subtype. METRNL+ is in an early stage during chondrocyte differentiation, and RegC-B is in an intermediate state before chondrocyte dedifferentiation. With cell differentiation, cell subtypes shift from genetic expression to extracellular matrix adhesion and collagen remodeling, and signal pathways shift from HIF-1 to Hippo. The 11 subtypes were finally classified as intrinsic chondrocytes, effector chondrocytes, abnormally differentiated chondrocytes and dedifferentiated chondrocytes. IHC was used to verify the presence and distribution of each chondrocyte subtype.

**Conclusion:**

This study screened two new chondrocyte subtypes, and a novel classification of each subtype was proposed. METRNL+ subtype is in an early stage during chondrocyte differentiation, and its transcriptomic characteristics and specific pathways provide a foundation for cartilage regeneration. EC-B, PRG4+ RegC-B, and FC are typical subtypes in the OA group, and the HippO-Taz pathway enriched by these cell subtypes may play a role in cartilage repair and OA progression. RegC-B is in the intermediate state before chondrocyte dedifferentiation, and its transcriptomic characteristics may provide a theoretical basis for intervening chondrocyte dedifferentiation.

## 1 Introduction

Osteoarthritis (OA) is one of the most common chronic diseases that cause disability in the elderly. More than 200 million people are affected by OA, which brings a great social burden (1). Traditionally, OA pathological changes are mainly the degeneration and loss of peri-articular cartilage and abnormal proliferation of subchondral bone (2). Therefore, clarifying the physiological and pathological characteristics of chondrocytes can provide the most direct reference for seeking ideal seed cells for cartilage regeneration (3). However, the role of OA chondrocytes in disease progression and the role of normal chondrocytes in cartilage regeneration have not been fully studied.

As a connective tissue, articular cartilage serves as lubricating and absorbing force via extracellular matrix (ECM), which accounts for about 95% volume of the cartilage, and is mainly composed of proteoglycans, collagen and water (4, 5). The cross-linking of proteoglycan and collagen is the key process to absorb force. Proteoglycan is thought to contribute more to absorbing force due to its binding to water (6), while collagen is considered to be more helpful in maintaining the integrity of cartilage tissue because of its cross-linking properties (7). Cartilage tissue can be divided into superficial, radial, deep, and calcified layers. In normal cartilage, the high percentage of collagen in the surface layer is beneficial to cope with the shear force, and the high content of proteoglycan in the radial layer and deep layer helps to cope with the pressure from the surface layer (8). The appearance of cartilage surface lesions in the early stage of OA indicates that collagen is one of the early stages of cartilage destruction (9). Account for around 5% volume of cartilage, chondrocytes maintain the turnover of ECM mainly through the balance of synthesis and catabolism. The imbalance of this process and the change of chondrocyte matrix gene expression profile are important factors causing cartilage tissue degeneration, and also the key targets in achieving cartilage regeneration (10–12). However, the functional heterogeneity of chondrocyte subtypes and its relationship to cellular synthesis and cartilage regeneration have not been adequately classified.

Single-cell RNA sequencing (scRNA-seq) characterizes the chondrocyte subtypes with different functional properties by clustering the transcriptomes of individual cells. Our previous scRNA-seq studies of cartilage from rheumatoid arthritic knee (13) and healthy ankle (14) identified two immune-associated chondrocyte subtypes and generated healthy ankle cartilage atlas, respectively. Tang et al. (15) conducted scRNA-seq on chondrocytes from OA cartilage and identified chondrocyte subsets, such as fibrochondrocytes (FC), effector chondrocytes (EC), homeostasis chondrocytes (HomC), regulatory chondrocytes (RegC), etc, for the first time. However, because of the small number of included chondrocytes, they did not analyze cartilage regeneration and OA related chondrocyte subtypes.

In order to screen chondrocyte subtypes related to cartilage regeneration and OA, we conducted scRNA-seq on a large number of chondrocytes from OA and normal femoral cartilage of the knee. This study aims to explore potential OA and cartilage regeneration associated chondrocyte subsets and supplement and verify previous studies. In addition, this study aims to deeply understand the differentiation sequence of each chondrocyte subtype, and explore the branching point of chondroblast subtype and abnormal differentiation subtype. Lastly, this study hopes to provide a theoretical basis for further exploring the pathogenesis of OA and seed cells for cartilage regeneration.

## 2 Methods

### 2.1 Volunteer screening and sample selection

**Figure 1:**
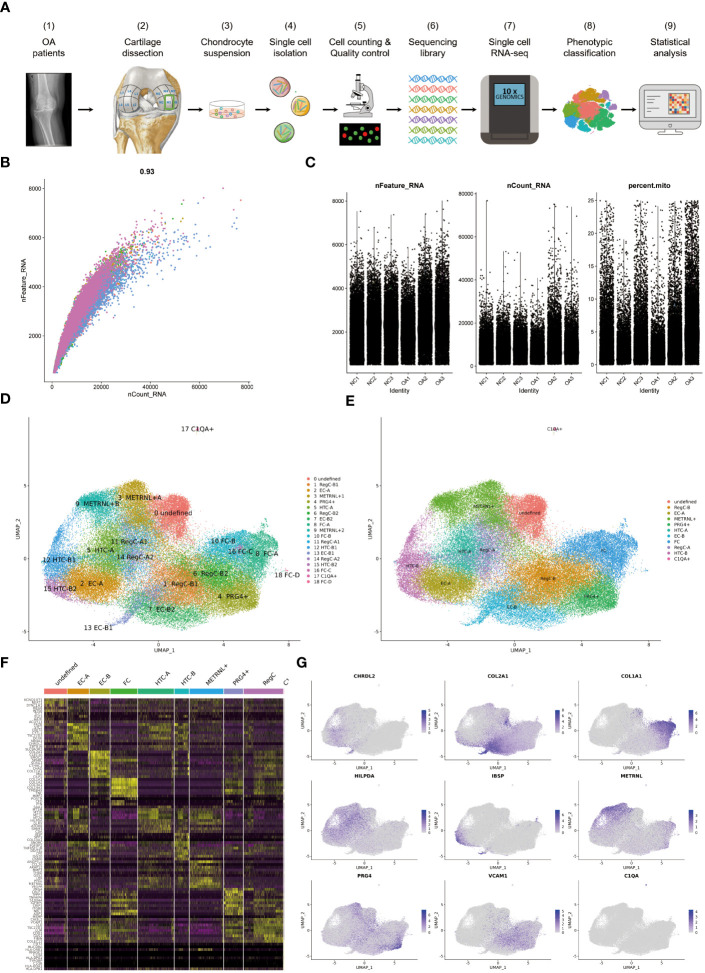
The process of scRNA-seq and the results of cell annotation **(A)** Flow chart of the experimental strategy. It includes sampling from femur of NC and OA, single cell isolation, cell counting and quality control, sequencing library preparation, single cell RNA-seq, phenotypic classification and statistical analysis. **(B)** Coefficient for nCount_RNA and nFeature_RNA **(C)** Parameters for screening nCount_RNA, nFeature_RNA and mitochondrial genes **(D)** Umap of all cells colored according to cell clusters. The annotation results of 19 clusters are listed on the right. **(E)** Umap of all cells colored according to cell subtypes **(F)** Heat map showing scaled differently expressed genes for each cell type defined in **(E)**. Specific representative genes in each cell type are listed along the left of heat map. **(G)** umap of all cells according to indicated marker genes for each cell subtype.

Samples were obtained from OA patients who underwent TKA and normal knee joint population for corpse donation. The inclusion criteria for patients were as follows: a diagnosis of primary osteoarthritis of the knee with K-L grade III or IV. For knee joints donated from corpses, samples were evaluated as healthy according to the Modified Outerbridge Classification before being included in this study. Exclusion criteria included genu valgus or genu varus ≥ 20°, inflammatory arthritis; gouty arthritis, congenital knee dysplasia and a history of previous knee surgery. The full layer of cartilage deep to the bone surface was obtained for scRNA-seq. Finally, we obtained 3 NC samples and 3 OA samples respectively. Next, we performed cell isolation, cell counting and quality control, scRNA-seq and data analysis (**Figure 1A**). This study was approved by the institutional ethics review board at the Affiliated Hospital of Qingdao University and all donors signed written informed consent.

### 2.2 Tissue dissociation and preparation of single-cell suspensions

Fresh cartilage tissue was transferred into a sterile culture dish containing phosphate-buffered saline (PBS) on ice, and cut it into 0.5 mm3 pieces. These pieces were washed with PBS twice and dissociated into single cells in dissociation solution (0.2% collagenase II 0.25% Trypsin-EDTA) in 37°C water bath with shaking for 20 min at 100 rpm. Digestion was terminated with PBS containing 10% fetal bovine serum (FBS).The resulting cell suspension was filtered by passing through 70–30 um stacked cell strainer and centrifuged at 300g for 5 min at 4°C. The cell pellet was added 100μl Dead Cell Removal MicroBeads (MACS 130–090-101) to remove dead cells using Miltenyi ® Dead Cell Removal Kit (MACS 130–090-101). Then, it was resuspended and certifuged twice at 300 g for 3 min at 4°C. The overall cell viability was confirmed by trypan blue exclusion, which needed to be above 85%. Single cell suspensions were counted using a Countess II Automated Cell Counter with a concentration of 700–1200 cells/μl.

### 2.3 10× Genomics scRNA-seq library preparation and sequencing

Single-cell suspensions were loaded to 10x Chromium to capture single cell according to the manufacturer’s instructions of 10x Genomics Chromium Single-Cell 3’ kit (V3). Steps of cDNA amplification and library construction were performed according to the standard protocol. Libraries were sequenced on an Illumina NovaSeq 6000 sequencing system (paired-end multiplexing run,150bp) by LC-Bio Technology co.ltd., (HangZhou, China) at a minimum depth of 20,000 reads per cell.

### 2.4 Bioinformatics analysis

Sequencing results were demultiplexed and converted to FASTQ format using Illumina bcl2fastq software (version2.20). We used Cell Ranger (https://support.10xgenomics.com/single-cell-geneexpression/software/pipelines/latest/what-is-cell-ranger, version 3.1.0) to analyze the quality of the original sequencing data of each sample and compared them with the reference genome (Ensembl genome GRCh38/GRCm38 reference genome). The analysis results of Cell Ranger were loaded into Seurat software (version3.1.1) for dimensional reduction and cluster analysis. Quality control was performed strictly. Firstly, the gene was detected in at least 3 cells were included. Secondly, the number of genes expressed in a single cell must be more than 500. Thirdly, the number of UMI must be greater than or equal to 500. Fourthly, the percent of mitochondrial-DNA derived gene-expression should be less than 25%.

To visualize the data, we further reduced the dimensionality of filtered cells using Seurat and used t-SNE to project the cells into 2D space. The steps included: using the Log Normalize method of the “Normalization” function of the Seurat package to calculated the expression value of genes; using the normalized expression value to perform PCA (Principal component analysis) analysis; using the top 10 PCs to do clustering and t-SNE analysis; selecting weighted Shared Nearest Neighbor (SNN) graph-based clustering method to find clusters. Marker genes for each cluster were identified with the Wilcoxon rank-sum test (default parameters is “bimod”: Likelihood-ratio test) via the FindMarkers function in Seurat. In addition, the gene expressed in more than 10% of the cells in a cluster and the gene expression multiple of log2FC ≥ 0.26 was identified as a marker gene. Differentially expressed genes in one cluster between HLB and LLB were also identified via the FindMarkers function in Seurat. Adjusted P value <0.05 and |log2 FC|>0.26 were set as cutoffs.

### 2.5 Gene ontology and Kyoto encyclopedia of genes and genomes pathway enrichment analysis

GO enrichment analysis provides significant GO terms by comparing differentially expressed genes with the whole gens of genome background (http://www.geneontology.org/). These significant GO terms (p-value < 0.05) provide clues for understanding the possible biological functions of cell population.

Genes usually interact with each other to play roles in certain biological functions. Pathway- based analysis helps to further understand biological functions of genes. Kyoto Encyclopedia of Genes and Genomes (KEGG) is the major public pathway-related database. Pathway enrichment analysis identified significantly enriched metabolic pathways or signal transduction pathways by comparing differentially expressed genes with the whole genome background genes.

### 2.6 Identification of different cell types

We further selected the top 10 genes as the marker genes according to differentially expression genes in each cluster. Then the distribution of each marker gene was demonstrated by using heat map, bubble diagram and umap. By comparing our marker genes and functional enrichments with that obtained from previous studies related to cartilage, we identified the cell type of each cluster.

### 2.7 Pseudotime analysis and RNA velocity analysis

Pseudotime analysis and trajectory construction were conducted by Monocle (http://cole-trapnell-lab.github.io/monocle-release/, version 2). The generated Seurat object was transformed into Monocle object with the “GetAssayData” and “newCellDataSet” function. The “orderCells” function in Monocle package arranged cells along a time axis to present their suggested position in a differential trajectory. The parameter of root_state was defined as ProC. The identified trajectory was colored by cell types or clusters to show the development pathway during chondrocyte differentiation.

### 2.8 Single-cell regulatory network inference and clustering

Seurat objects with annotations were imported to run Scenic (version 0.9.1). Use GENIE3 to extrapolate gene regulatory networks from gene expression data. The TF targeting relationship with the gene was verified with the help of RcisTarget software. Then, cells with active gene regulatory networks were identified using AUCell software, and regulons were scored for their Area Under Curve (AUC) by binarizing specific regulons. The RSS score of the TF was obtained by the calcRSS and ranked by value to determine the Top TF for each cell type. The differentially expressed transcription factors were visualized using the pheatmap function.

### 2.9 Immunohistochemical assays and cellular immunofluorescence detection

The immunohistochemical staining of trimmed articular cartilage tissue includes: fixing in 4% buffered paraformaldehyde for 48 hours, decalcifying with EDTA for about 4 weeks, embedding and sectioning in paraffin, dewaxing and rehydrating paraffin sections with xylene and gradient alcohol, blocking endogenous peroxidase activity with 3% H2O2 for 10 minutes, serum sealing with 3% BSA for 30 minutes at room temperature, antigen retrieval with pepsin digestion, primary antibody incubation overnight at 4°C, corresponding secondary antibody incubation for 50 minutes at room temperature, DAB color reaction and counterstaining nucleus with hematoxylin stain solution for about 3 minutes. Upright microscope (NIKON Eclipse ci) and imaging system (NIKON digital sight DS-FI2) were applied to enlarge and take photos for the histological section using white light. The widely accepted German semi-quantitative scoring system was used, taking into account the range of staining strength and staining area. Staining intensity: 0, no coloring; 1, light yellow; 2, brown-yellow; 3, brown- brown. Stained area: 0, < 5%; 1, 5% -25%; 2, 25%-50%; 3, 51%-75%; 4, >75%. These two scores were multiplied together as the final score. Comparative analysis of IHC staining scores for each layer of cartilage tissue was performed using the Kruskal-Wallis test for within-group differences and the Wilcoxon rank sum test for between-group differences.

The immortalized human chondrocytes (iCell Bioscience Inc, Shanghai) were passaged onto glass slides in a 6-well plate and grown to an experimental usable state. After washing with PBS buffer, the cells were fixed with 4% paraformaldehyde for 15 minutes. ANGPTL4 and CRLF1 primary antibodies were added separately and incubated overnight at 4°C to enhance antibody-cell interactions. After washing to remove unbound primary antibodies, corresponding secondary antibodies were added and incubated for 1 hour at 4°C. Following another wash to remove unbound secondary antibodies, anti-fade mounting medium was added to prevent signal fading. The slides were covered with glass coverslips, and fluorescence imaging was performed using a fluorescence microscope.

### 2.10 Statistical analysis

SPSS version 23.0 (IBM Inc., Armonk, NY, USA) was used to compare the difference in cell numbers between the NC group and OA group. T test was applied for testing, and P < 0.05 was considered to be statistically significant.

## 3 Results

### 3.1 Single-cell profiling and identification of femoral cartilage chondrocytes

75,104 cells were analyzed and grouped into clusters 0–18 (**Figure 1D**). The condition for quality control is shown in **Figures 1B, C**. The 19 clusters were then summarized and annotated into 11 distinct cell subtypes, including EC-A, EC-B, HTC-A, HTC-B, RegC-A, RegC-B, FC, METRNL+ subtype, PRG4+ subtype, C1QA+ subtype and an undefined subtype (**Figure 1E**). **Figure 1F** presents some highly expressed DEGs in each subtype. The marker genes for subtypes discussed in this study are shown in **Figure 1G**, which shows that CHRDL2 for EC-A, COL2A1 for EC-B, HILPDA for HTC-A, IBSP for HTC-B, VCAM1 for RegC-B, METRNL and ANGPTL4 for MERNL+subtype, PRG4 and CRLF1 for PRG4+ subtype.

**Figure 2:**
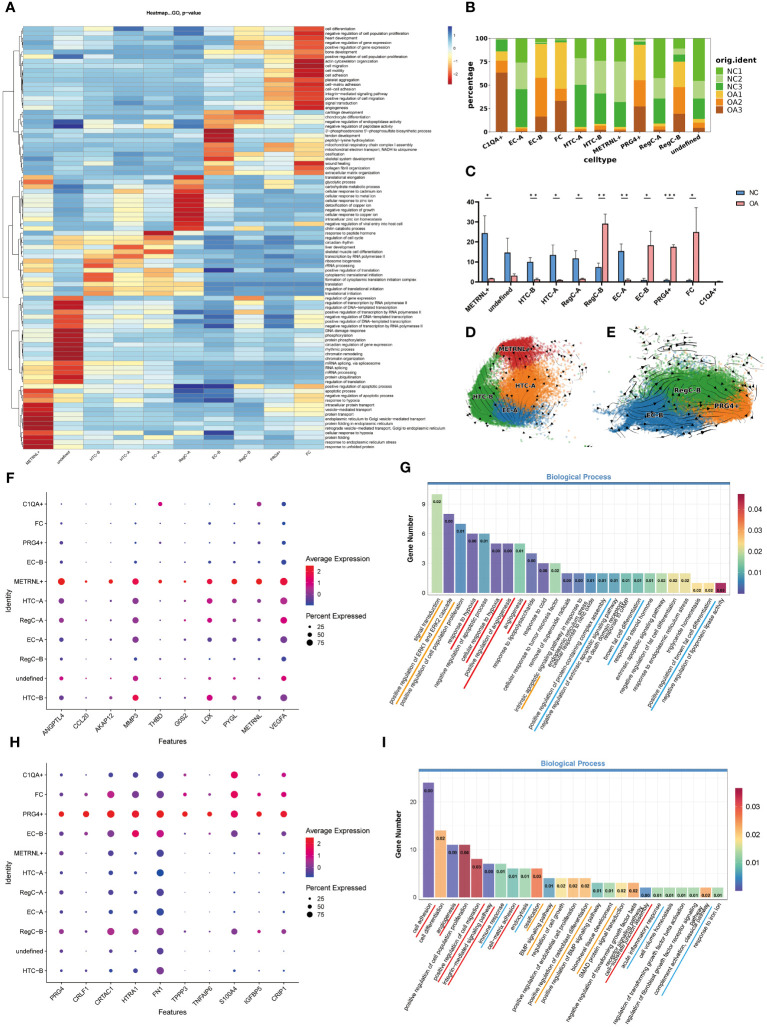
Enrichment analysis and proportion of each subtype, and RNA velocity analysis of two newly identified subtypes **(A)** GO enrichment analysis of each subtype. The top 20 biological process terms for each subgroup based on p-value were summarized and visualized by heatmap according to -log10p values. **(B)** The proportion of each subtype in each sample **(C)** t-test for the proportion of the number of subtypes in each sample. *p < 0.05, **p <0.01, ***p <0.001 **(D)** RNA velocity analysis of METRNL+ subtype, HTC-A, HTC-B, and EC-A. Arrows show the direction and rate of cellular movement. **(E)** RNA velocity analysis of EC-B, RegC-B, and PRG4+ subtypes. **(F)** The top 10 DEGs of METRNL+ subtype based on log2FC. **(G)** GO enrichment analysis of METRNL+ subtype. The gene with Log2FC*PCT1/PCT2≥1.5 in this subtype was applied to enrichment analysis. **(H)** The top 10 DEGs of PRG4+ subtype based on log2FC. **(I)** GO enrichment analysis of PRG4+ subtype. The gene with Log2FC*PCT1/PCT2≥1.5 in this subtype was applied to enrichment analysis.

**Figure 3:**
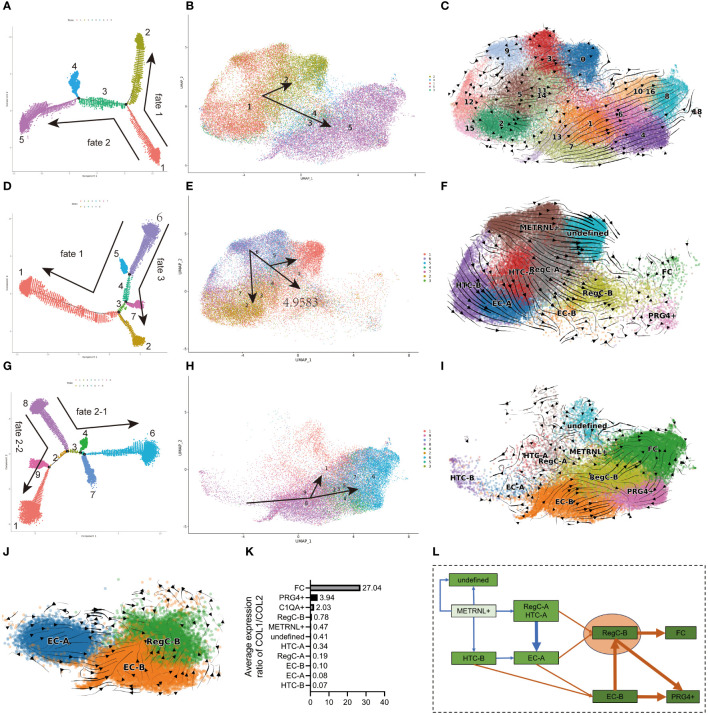
Differentiation sequence of chondrocyte subtypes **(A, D, G)** pseudo-time analysis of all cells, NC group and OA group. The dots in each color represents each state, and each state is named numerically. The direction of the arrow represents the direction of the pseudo-time. **(B, E, H)** umap visualization of state in **(A, D, G)**. The meanings of the colors, numbers, and arrows correspond to the **(A, D, G)**, respectively. **(C, F, I, J)** RNA velocity analysis of EC-A, EC-B, and RegC-B in all cells, NC group, and OA group cells. **(K)** Relative expression levels of COL1/2 subtypes **(L)** Summary of the differentiation sequence of each subtype. The arrows represent the differentiation direction.

Besides referring to marker genes reported in the literatures, we also considered the results of enrichment analysis (**Figure 2A**) to annotate cell subtypes. We summarized the top 20 terms ranked by p-value of each subtype in the biological process of GO enrichment analysis and presented them by heatmap. It was found that the subtypes that have a higher percentage in the NC group (**Figures 2B, C**) mainly presented hierarchical changes around GO terms related to the genetic expression process (**Figure 2A**). Although marker gene for the undefined subgroup was not screened successfully, the results of enrichment analysis showed that it was more prominent in the gene and chromatin damage repair. Although the functions including transcription, translation, protein modification, and transport for the remaining five subtypes were all significant, their specificities were different. METRNL+ subtype tended to play an important role in protein processing and transport, HTC-B was involved in endoplasmic reticulum translation, and HTC-A is preferentially enriched in transcription and ribosome processing. EC-A exhibited reactive activity, including biological processes such as response to hormones, regulation of the cell cycle, and differentiation into skeletal muscle cells. The GO enrichment results of RegC-A (cluster 11 and 14) are partly similar to the metal ion associated chondrocytes (MIRC) mentioned in the previous study of our team, which was enriched in response to metal ions (14). However, in combination with the differentiation sequence for RegC-A and RegC-B suggested by RNA velocity analysis shown in **Figure 3F**, and the specific expression of RegC marker genes such as CHI3L1, CHI3L2 and VCAM1 mentioned by Tang et al (15) in cluster11 and 14, we annotated them as RegC-A.

The subtypes that have a higher percentage in the OA group (**Figures 2B, C**) were mainly hierarchical enriched in GO terms related to damage repair (**Figure 2A**). EC-B was mainly enriched in responding to injury, such as ECM and collagen remodeling. Chondrocytes that specifically express COL1 have been considered as a sign of chondrocyte dedifferentiation, in the current study, FC owned this characteristic (**Figure 3K**). FC was preferentially enriched in cell migration and adhesion, ECM adhesion, and angiogenesis. RegC-B was mainly enriched in chondrocyte differentiation and development. Although the p-value was slightly lower, the GO terms of the PRG4+ subtype are similar to those of FC. In addition, we identified the GO items related to osteochondral biological function (**Supplementary Figure 1A**) with a p value of less than 0.05. The results showed that EC-B, RegC-B, PRG4+ and FC were more enriched in osteogenic function, cartilage differentiation and development than those in the subtypes that have a higher percentage in the NC group.

To compare the difference for each cell subtype between the NC and OA groups, we analyzed the proportion of cell subtypes in each sample (**Figures 2B, C**). The results showed that the proportion of EC-A, HTC-A, HTC-B, RegC-A and METRNL+ subtypes in the NC group was higher than that in the OA group (p<0.05), while the proportion of EC-B, RegC-B, FC and PRG4+ subtypes in NC group was lower than that in OA group (p<0.05). Considering the results of pseudo-time and RNA velocity analysis (**Figure 3**), we classified HTC-A, HTC-B, RegC-A, and METRNL+ subtype as inherent chondrocytes (InC), EC-A, and EC-B are classified as effector chondrocytes (EC), RegC-B and PRG4+ subtype as abnormally differentiated cells, and FC as dedifferentiated chondrocytes.

### 3.2 Biological characteristics METRNL+ and PRG4+ subtypes

As mentioned above, the number of METRNL+ subtype was much more in the NC group compared with the OA group, and its function such as the genetic expression process was particularly prominent. RNA velocity analysis of functionally similar subtypes (**Figure 2D**) showed that METRNL+ had the most active RNA metabolism, suggesting that it may be in the early stage during the differentiation process. Further exploration of the genetic expressional characteristics of METRNL+ subtype showed that besides METRNL, genes such as ANGPTL4, MMP3, LOX, PYGL, VEGFA, etc were also specifically highly expressed (**Figure 2F**, **Supplementary Figure 2A**). To explore the functional characteristics of METRNL+ subtype, DEGs of this subtype were further screened for more specific genes based on the results of Log2FC*PCT1/PCT2≥1.5 and then transferred to GO enrichment analysis (**Figure 2G**). The results suggested that this subtype is preferentially enriched in responding to hypoxia, angiogenesis, protein assembly, and differentiation into brown fat cells. In addition, signal transduction and apoptosis may be active in this subtype. We sorted out GO terms associated with bone and cartilage biological functions (**Supplementary Figure 1A**) and GO terms related to the METRNL gene (**Supplementary Figure 1B**) with a subset having a p-value less than 0.05. Our results demonstrate that, although this subtype exhibits enrichment in osteogenesis in METRNL+, the EC-B, RegC-B, PRG4+, and FC subgroups show a more pronounced enrichment in osteogenic functional terms based on p-value and rich factor. Notably, the METRNL+ subgroup displays a significant enrichment in chondroitin sulfate synthesis function. In summary, this subtype may play an important role in cartilage tissue maintenance and renewal.

We applied the same method to the PRG4+ subtype which has a lower proportion in the NC group compared with the OA group. RNA velocity analysis (**Figure 2E**) showed that RNA metabolism of PRG4+ subtype was slower than that of EC-B and RegC-B, suggesting that PRG4+ should be in a late stage during differentiation. PRG4+ subtype was preferentially enriched in GO terms related to cellular and tissue adhesion (**Figure 2I**), 24 genes such as FN1 and TNFAIP6 with Log2FC*PCT1/PCT2≥1.5 (**Figure 2H**, **Supplementary Figure 2C**) were associated with these biological processes. In addition, 15 genes, including PRG4, CRIP1 and CD55, were enriched in GO terms related to inflammation (**Figure 2I**). Furthermore, this subtype was also associated with ECM ossification, fibrogenesis, and TGFβ process (**Figure 2I**). In summary, this subtype may respond to injury and inflammation, and own ECM fibrogenesis and osteogenesis potential.

### 3.3 Pseudo-time and RNA velocity reveal the differentiation order of cell subtypes

We performed pseudo-time (**Figures 3A, D, J**) and RNA velocity analysis (**Figures 3C, F, I**) on all cells, NC group and OA group, respectively, and the state determined by pseudo-time analysis was further visualized by umap plot (**Figures 3B, E, H**). The differentiation sequence of each chondrocyte subtype is presented in **Figure 2K**. In detail, for all cells, cells in state1(METRNL+ chondrocyte, HTC, and EC-A) differentiated to both fate1 and fate2 directions, which represented state2 (undefined subtype) and state5 (EC-B, RegC-B, FC, PRG4+ chondrocyte), respectively (**Figures 3A–C**). This suggested a trend that the cells in the NC group differentiated into the OA group. The results of RNA velocity analysis of all cells also corresponded to this trend (**Figure 3C**).

Originating from state 6 (METRNL+ chondrocyte), cells in the NC group also differentiated to fate 3 (state 2, EC, and HTC) besides fate 1(**Figures 3D–F**). Consistent with the results of pseudo-time analysis, the RNA velocity analysis is presented in **Figures 2D** and **3F** showed that the METRNL+ subtype was in an early stage during differentiation and differentiated to EC-A, RegC-B, and undefined subtype, respectively. As shown in **Figures 3G–I**, cells in the OA group originated from state8 (HTC-B and EC-B) and were further differentiated to FC via RegC-B. In combination with the results shown in **Figure 2E**, RegC-B also showed a tendency to differentiate to the PRG4+ subtype. In order to further clarify the differentiation relationship among EC-A, EC-B, and RegC-B, we conducted RNA velocity analysis on these cells (**Figure 3J**), and the results suggested that EC-A and EC-B could both differentiate to RegC-B, and EC-A tended to differentiate to EC-B.

In summary (**Figure 3L**), first, the METRNL+ subtype was in an early stage during differentiation, and the METRNL+ subtype in the NC group mainly differentiated into three directions: EC-A, RegC-B and undefined subtype. Second, FC was in a late stage that exhibited a dedifferentiated character, RegC-B was the major origin of FC, and EC-B was the main origin of RegC-B. In addition, EC-A and HTC-B were likely to be the main origins of EC-B. Last, part of EC-B and RegC-B were likely to differentiate to the PRG4+ subtype. We used the relative expression levels of COL1/COL2 to determine the dedifferentiation degree of each subtype (**Figure 3K**), which corresponded to the results of pseudo-time and RNA velocity analysis.

### 3.4 Functional changes during cell differentiation

**Figure 4:**
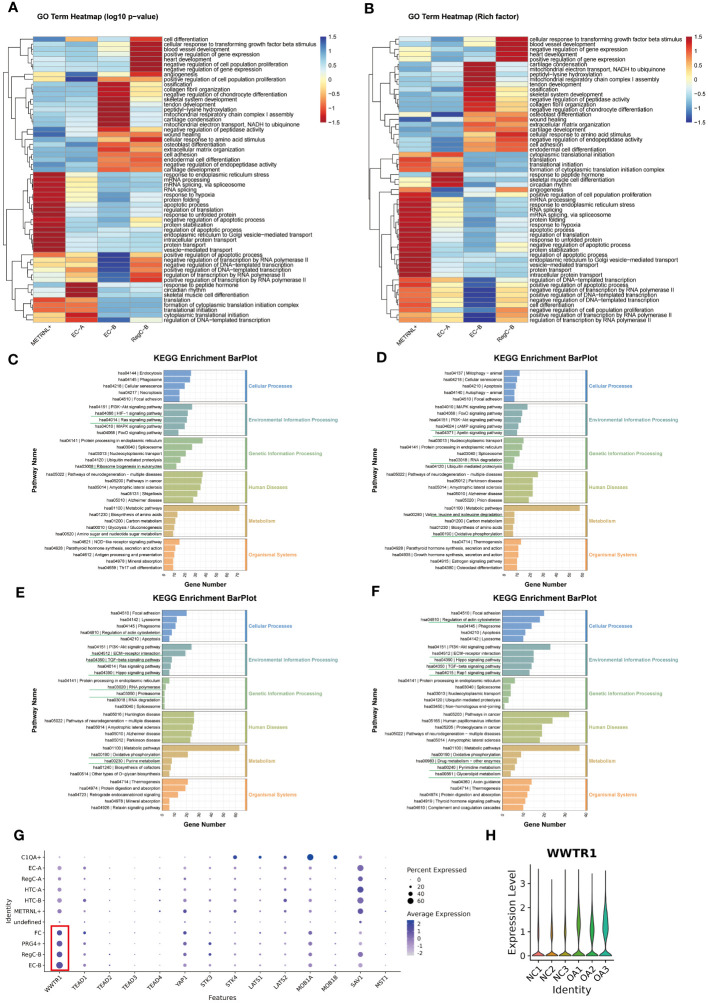
Changes in enrichment analysis during cell differentiation **(A, B)** GO enrichment analysis of each subtype. The top 20 biological process terms based on p-value for each subtype were summarized and visualized by heatmap according to either the -log10p value **(A)** or the -log10Rich factor **(B)**. **(C–F)** KEGG pathway enrichment analysis of METRNL+, EC-A, EC-B and RegC-B. The bottom horizontal axis represents the number of genes enriched into the each pathway. **(G)** Expression of important downstream target genes of Hippo signaling pathway in each subtype. The size of the circle represents the proportion of cells in the cell subtype expressing the gene, and the color of the circle represents the average gene expression. **(H)** Expression of WWTR1 in each sample.

To explore the functional evolution from intrinsic chondrocytes to abnormally differentiated chondrocytes, we performed transcriptomic comparisons and enrichment analysis on METRNL+ subtype, EC-A, EC-B, and RegC-B (**Figure 4**). We summarized the top 20 enrichment terms based on the p-value of biological processes in the GO enrichment analysis (**Figures 4A, B**), which showed that the METRNL+ subtype focused on RNA splicing, protein translation and transport, and apoptosis. EC-A preferred to participate in peptide hormone response, skeletal muscle cell differentiation, circadian rhythm, cytoplasmic translation initiation, and transcriptional regulation. EC-B were enriched in ECM (including collagen) remodeling and ossification, chondrocyte coagulation, and cell differentiation. RegC-B were preferentially enriched in the TGF-β pathway, blood vessel formation and development, wound healing, and cell adhesion.

The results of KEGG analysis suggested that the METRNL+ subtype was enriched in HIF-1 and Ras signaling pathway, ribosome biosynthesis, amino sugar and nucleotide sugar metabolism, and glycolysis (**Figure 4C**). EC-A (**Figure 4D**) were involved in cAMP and Apelin signaling pathways, RNA degradation and degradation of valine, leucine, and isoleucine, and oxidative phosphorylation. EC-B preferred to participate in cytoskeletal regulation, Hippo, TGF-beta, and ECM-receptor interaction signaling pathways, RNA polymerase and proteasome, oxidative phosphorylation, and purine metabolism-related items (**Figure 4E**). To explore the targets of the Hippo signaling pathway, we compared the expression of all downstream target genes among all subtypes (**Figure 4G**) and all samples (**Figure 4H**), respectively, and found that the TAZ target (WWTR1) was more highly expressed in EC-B, RegC-B, PRG4+, FC, and OA samples. Therefore, we speculated that HiPO-TAZ should be one of the important ways that induce or against OA. Unlike EC-B, RegC-B was also enriched in the Rap1 signaling pathway, pyrimidine and triglyceride metabolism, drug metabolism and other related terms (**Figure 4F**).

In general, with the process of METRNL+ differentiation to RegC-B, the priority of protein synthesis and apoptosis gradually decreased. However, the functions of collagen synthesis and ECM ossification, chondrocyte coagulation and differentiation, cell adhesion and wound healing, angiogenesis and development have gradually increased in priority.

### 3.5 Differences in transcriptional regulation during cell differentiation

**Figure 5:**
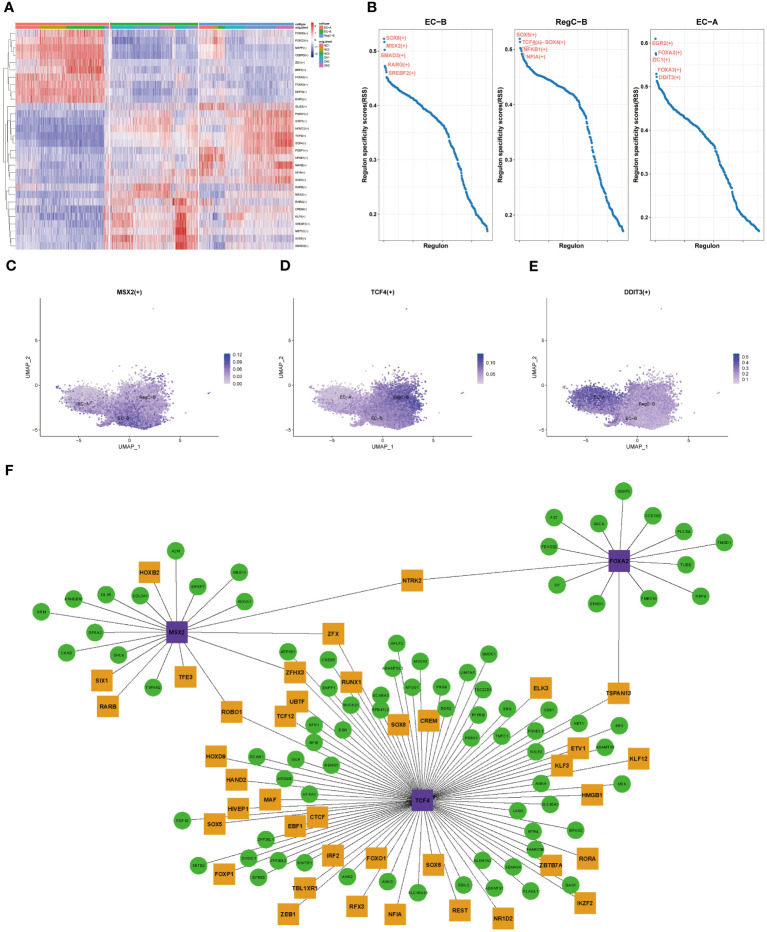
Transcriptional regulation of chondrocyte subtypes **(A)** Heatmap of specific transcriptional factors of EC-A, EC-B, and RegC-B **(B)** RSS scores for specific transcription factors of EC-A, EC-B and RegC-B. **(C–E)** umap plot for key transcription factors of EC-A, EC-B, and RegC-B. **(F)** Gene regulatory network of specific transcription factors. The purple genes are the screened key transcription factor. The green and yellow genes are the highly expressed DEGs in the chondrocyte subtypes, and the yellow genes means that the gene itself is a transcription factor.

To clarify the evolution of transcriptional regulation from EC to abnormally differentiated chondrocytes, we performed transcriptional comparisons and functional enrichment analyses on EC-A, EC-B, and RegC-B (**Figure 5**). **Figure 5A** shows the top 10 specific transcription factors for each chondrocyte subtype. After RSS scoring, we obtained 5 prominent transcription factors for each subtype. Three significant transcription factors, MSX2 (EC-B), TCF4 (RegC-B), and DDIT3 (EC-A), were finally screened according to the expression levels of transcription factors based on umap plot (**Figures 5C–E**). To clarify the regulatory characteristics of the three transcription factors, we mapped the regulatory networks among the three transcription factors and DEGs of their subtypes.

### 3.6 The distribution of subtypes in cartilage tissue

**Figure 6:**
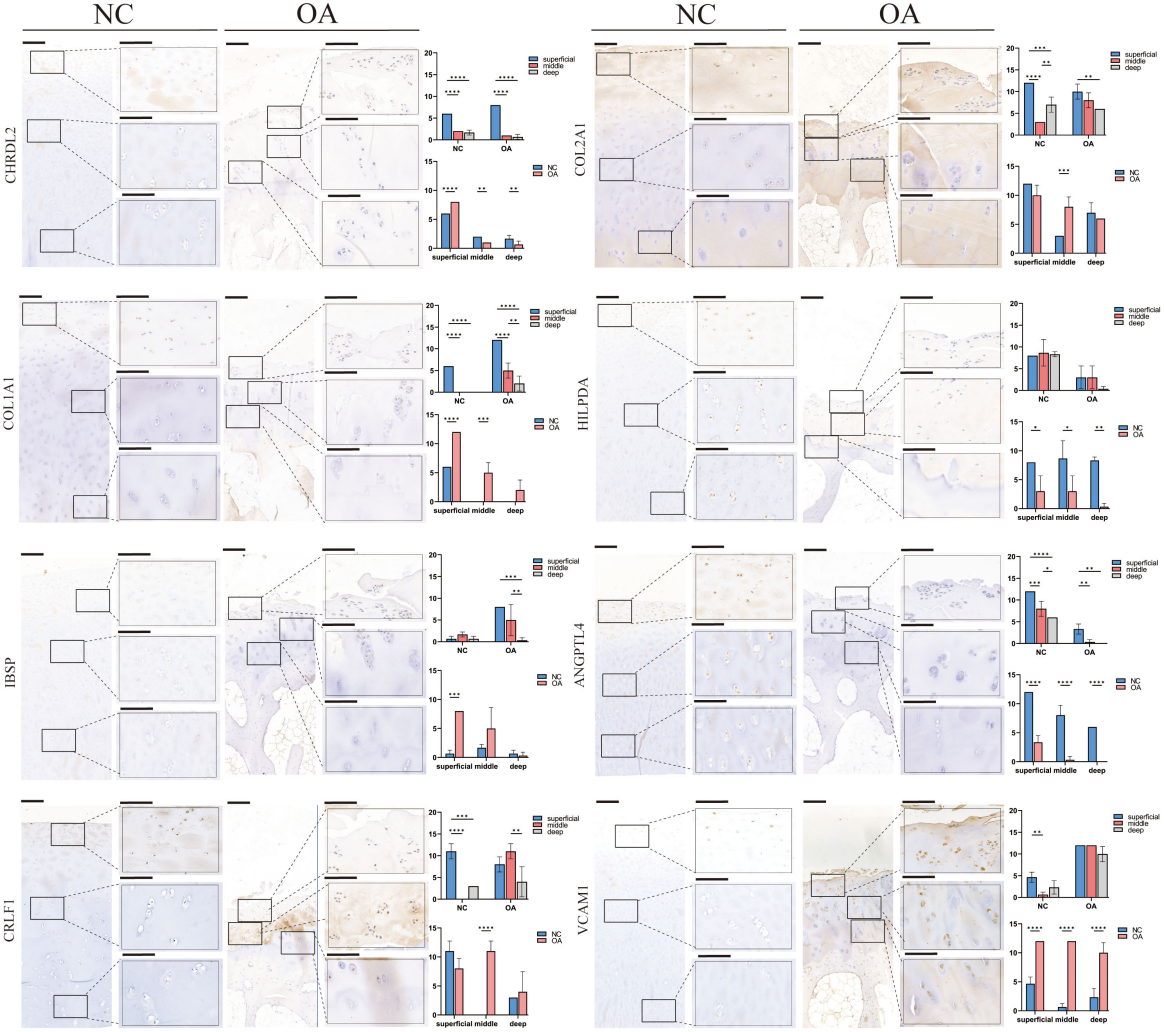
IHC results for specific markers for each cell type in different groups and layers. Scale bar, left, 200 μm; right, 50 μm. The scores of the indicated genes in the cartilage of both regions based on the immunohistochemistry assay are shown. *p<0.05, **p<0.01, ***p<0.001; ****p<0.0001; otherwise, not significant NC, normal femur cartilage; OA, OA femur cartilage.

Similar to the percentage of chondrocyte subtypes in the two groups of scRNA-seq, IHC also indicated that the number of EC-A, HTC-A, and METRNL+ chondrocytes in the NC group was more than that in the OA group (p<0.05), and the number of RegC-B and FC in OA group was higher than that in OA group (p<0.05) (**Figure 6**). In addition, compared with the NC group, the number of EC-B and PRG4+ chondrocytes were more only in the middle layer of cartilage from the OA group (p<0.05) (**Figure 6**). Moreover, compared with the intermediate and deep layers, EC-A, EC-B, FC, METRML+, and PRG4+ chondrocytes were more abundant in the shallow layer (**Figure 6**). To further validate the two newly proposed subtypes, we conducted immunofluorescence staining on human chondrocytes. The results revealed widespread expression of the differentially expressed genes in these subtypes within human chondrocytes (**Supplementary Figures 2B, C**).

## 4 Discussion

Chondrocytes are traditionally considered to have a poor regenerative ability and cannot be satisfactorily repaired after lesion. Recent studies tended to use stem cells and autologous chondrocytes in regenerating cartilage, but their efficacy remains to be further verified (16). This study screened METRNL+ cell subsets in the femoral cartilage of the knee for the first time. Although the role of METRNL in osteoblast function has been previously reported (17, 18), our results suggest that the osteogenic function is not the predominant function in the METRNL+ subtype. Its GO terms focus on functions such as protein synthesis and transportation, adding that representative genes of catabolism such as MMP3 are specifically expressed in this subtype, indicating that this subtype is a group of chondrocytes with active synthesis and catabolism process. The balance between synthesis and catabolism in chondrocytes is the driving force for cartilage tissue to maintain and renew its ECM (19, 20). Moreover, previous research has demonstrated the potential of METRNL in mitigating inflammation and pyroptosis in osteoarthritis (21), combined with METRNL+ subtype mainly comes from healthy cartilage, which suggest the cartilage protective effect of this subtype. Furthermore, pseudo-time analysis and RNA velocity analysis suggested that METRNL+ subtype belonged to early differentiated chondrocytes. In summary, the discovery of this subtype and its transcriptomic properties is expected to provide a new theoretical basis and practical direction for cell transplantation and cartilage regeneration.

The study conducted by Tang et al (15). mentioned that RegC might be involved in immune inflammatory response. A current study found that besides RegC, a group of chondrocytes with high expression of CRLF1, CD55, CRIP1, and PRG4 were also involved in the inflammatory response, and GO enrichment analysis showed that they tended to respond to acute inflammation. GO terms such as adhesion function of cells and extracellular matrix, osteogenesis and ossification regulation were also clearly significant in this subtype, which are closely related to the abnormal repair of cartilage injury (22–24). Therefore, it is expected to provide a new direction for preventing diseases including cartilage injury and OA by intervening in the abnormal repair of this subtype and verifying the mechanism for abnormal repair.

It is believed that EC is in the early stage during chondrogenic differentiation, and RegC is distributed throughout the chondrogenic differentiation process (15, 25). In this study, through pseudo-time and RNA velocity analysis, we suggested that the METRNL+ chondrocyte subtype should be distributed in a much earlier stage. EC owns a property of active compensatory, and its differentiation sequence may be after METRNL+ chondrocytes. RegC-B, as an intermediate state in the transition from nearly all OA and NC chondrocytes to FC, may be in an abnormal differentiation stage during the dedifferentiation process. COL1/2 is considered to be an important indicator of chondrocyte dedifferentiation (26), and the dedifferentiating degree of RegC-B in this study was between FC and EC. Therefore, the transcriptional regulation mechanism of RegC-B may provide a new direction for chondrocyte dedifferentiation.

Submerged in the joint fluid, chondrocytes are in a state of gradient hypoxia, and it has been found that the activation of the HIF-1 pathway, which is associated with hypoxia, stimulates the anabolism of chondrocytes (27, 28). In this study, the METRNL+ subtype was enriched in responding to hypoxia according to GO enrichment, and the HIF-1 pathway was also enriched in KEGG pathway, which further suggested the protective role of METRNL+ chondrocytes in the maintenance and regeneration of cartilage tissue. In addition, with the process of abnormally differentiation and dedifferentiation, the significant enriched pathway for RegC-B was transformed into the Hippo signaling pathway. Most scholars believe that the Hippo-YAP signaling pathway plays a protective role against (29, 30), but lack of reports on the role of the Hippo-TAZ pathway in the process of OA cartilage (31). The results of the current study suggest that Hippo-TAZ should be an important pathway to cause or against OA. Therefore, the mechanism Hippo-TAZ pathway on cartilage should be further explored.

Compared with previous studies, the current study conducted scRNA-seq on more chondrocytes and focused on the distal femur cartilage. In addition, the current study deeply analyzed each cell subtype in a customized way, speculated and classified the intrinsic chondrocytes, effector chondrocytes, abnormally differentiated chondrocytes, and dedifferentiated chondrocytes. Moreover, the current study explored the possible differentiation sequence and functional changing of the chondrocytes through pseudo-time and RNA velocity analysis, and proposed the potential state evolution of chondrocytes. Lastly, the typical cells of the NC and the OA groups and their transcriptomic characteristics were obtained via comparing the differences between the two groups, which is expected to provide potential targets for cartilage disease intervention and cartilage regeneration. The obvious differences between the two groups also provided straightforward results for future studies. However, the sample of mild cartilage lesion from OA was not included in this study, and the cellular atlas and transcriptomic characteristics of early OA may not be fully summarized.

## 5 Conclusion

METRNL+ is a typical subtype in the NC group, which is an early differentiation chondrocyte and may have a protective effect on cartilage tissue renewal and regeneration. EC-B, FC, PRG4+ and RegC-B cell subsets are typical cells in the OA group. The cartilage repair mediated by these subtypes is different from the renewal mechanism of ECM by normal chondrocytes, which may be the intrinsic factor of OA cartilage tissue degeneration. RegC-B is in the intermediate state before chondrocyte dedifferentiation, and its transcriptomic characteristics provide a theoretical basis for intervening chondrocyte dedifferentiation. HIF-1 is a typical pathway in normal chondrocytes, and the HiPO-TAz pathway may play a role in OA cartilage repair and OA progression.

## Data availability statement

The datasets presented in this study can be found in online repositories. The names of the repository/repositories and accession number(s) can be found below: PRJNA1105421(SRA).

## Ethics statement

The studies involving humans were approved by the Ethics Review Committee of the Affiliated Hospital of Qingdao University. The studies were conducted in accordance with the local legislation and institutional requirements. The participants provided their written informed consent to participate in this study. Written informed consent was obtained from the individual(s) for the publication of any potentially identifiable images or data included in this article.

## Author contributions

ZS: Conceptualization, Data curation, Formal Analysis, Investigation, Methodology, Software, Supervision, Validation, Writing – original draft, Writing – review & editing. MY: Conceptualization, Data curation, Investigation, Software, Methodology, Writing – review & editing. JW: Data curation, Methodology, Software, Conceptualization, Investigation, Writing – review & editing. HZ: Data curation, Methodology, Software, Writing – review & editing. XJ: Data curation, Methodology, Writing – review & editing. YX: Data curation, Methodology, Writing – review & editing. TW: Conceptualization, Investigation, Project administration, Resources, Supervision, Validation, Visualization, Writing – review & editing. TY: Conceptualization, Investigation, Project administration, Resources, Supervision, Validation, Visualization, Writing – review & editing.

## Supplementary material

Supplementary Figure 1 GO terms related to osteochondral biological functions and the METRNL gene. **(A)** GO terms related to osteochondral biological functions. The color gradient represents the range of p-values from 0 to 0.01, while GO terms with p-values ranging from 0.01 to 0.05 are uniformly indicated in gray **(B)** GO terms related to the METRNL in METRNL+ subtype.

Supplementary Figure 2 Immunofluorescence staining on human chondrocytes. **(A, C)** The top 10 DEGs of METRNL+ and PRG4+ subtype respectively based on log2FC. **(B, D)** Immunofluorescence staining of METRNL+ and PRG4+ subtype respectively. The three panels in the figure represent the immunofluorescence of antibodies (left panel), DAPI staining (middle panel), and the merged image of both (right panel), respectively. The images were captured at a magnification of 40X.
