# Supplementary figures and images for: Single-cell RNA sequencing reveals different chondrocyte states in femoral cartilage between osteoarthritis and healthy individuals
Source: Front Immunol. 2024 May 29;15:1407679. doi: 10.3389/fimmu.2024.1407679 (PMC11167083; doi:10.3389/fimmu.2024.1407679)

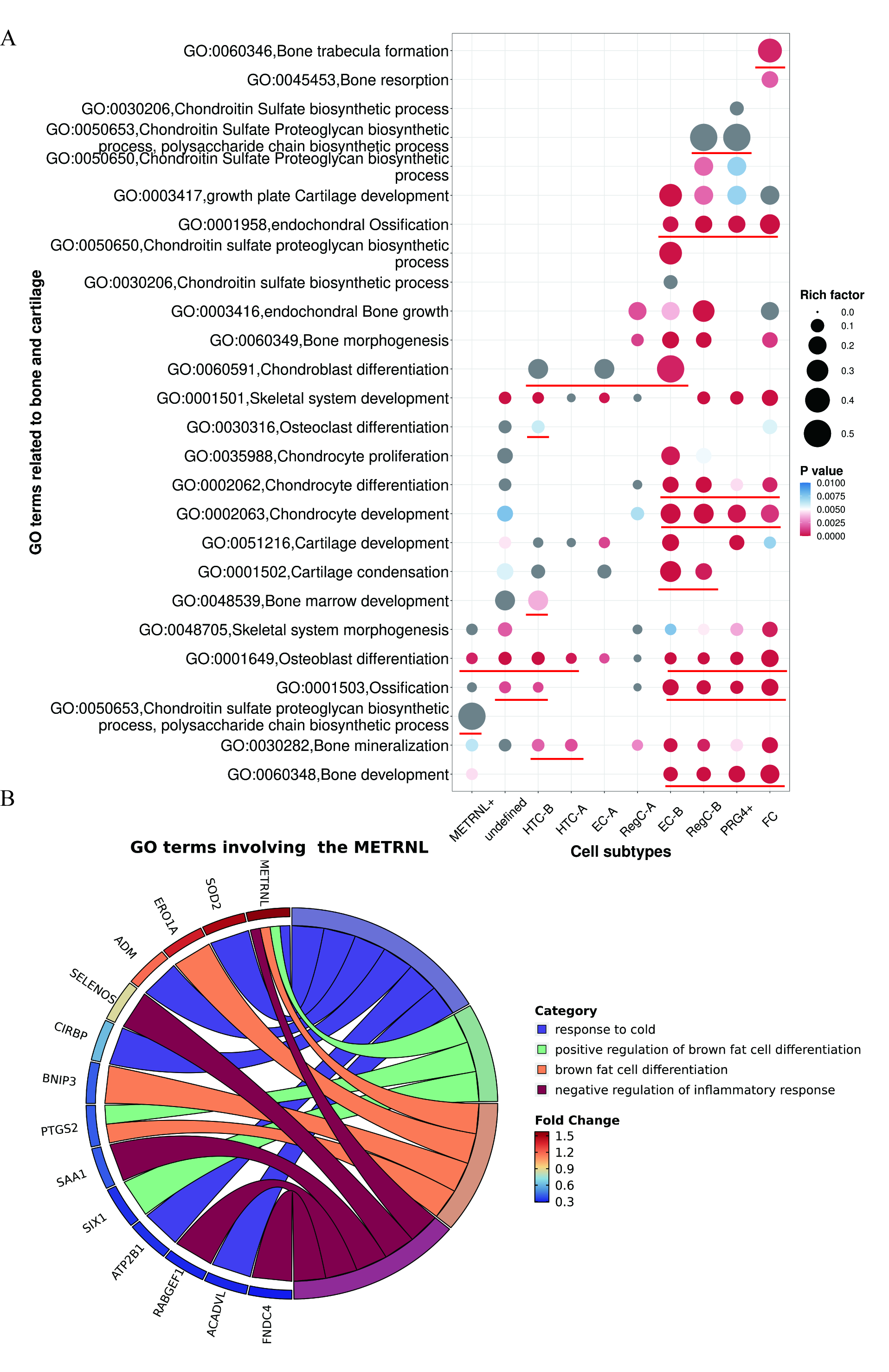

Supplement: Supplementary Figure 1 — GO terms related to osteochondral biological functions and the METRNL gene. (A) GO terms related to osteochondral biological functions. The color gradient represents the range of p-values from 0 to 0.01, while GO terms with p-values ranging from 0.01 to 0.05 are uniformly indicated in gray (B) GO terms related to the METRNL in METRNL+ subtype. [file Image_1.tif]

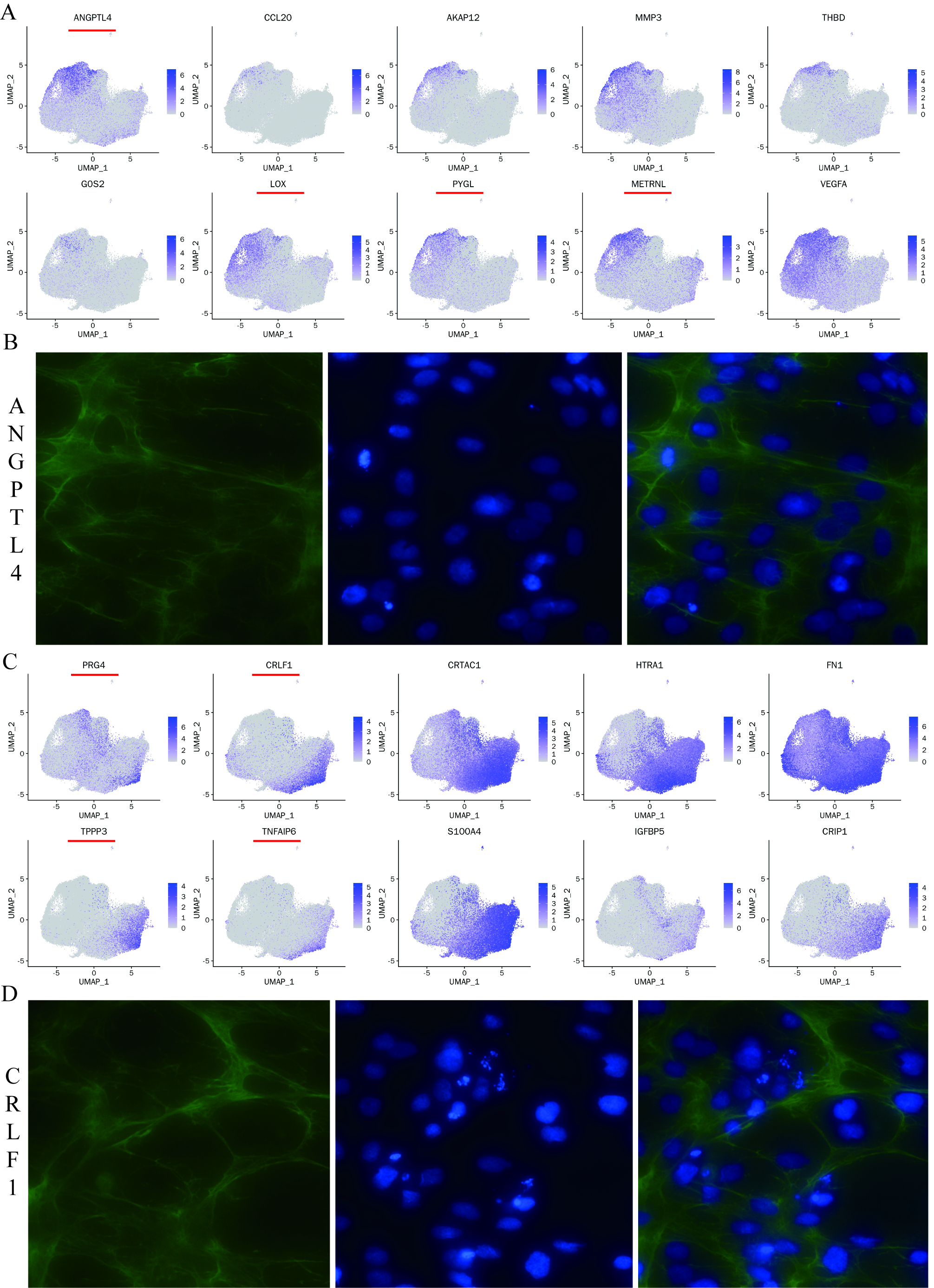

Supplement: Supplementary Figure 2 — Immunofluorescence staining on human chondrocytes. (A, C) The top 10 DEGs of METRNL+ and PRG4+ subtype respectively based on log2FC. (B, D) Immunofluorescence staining of METRNL+ and PRG4+ subtype respectively. The three panels in the figure represent the immunofluorescence of antibodies (left panel), DAPI staining (middle panel), and the merged image of both (right panel), respectively. The images were captured at a magnification of 40X. [file Image_2.tif]
